# Magnetic Resonance Imaging of the Clivus and Its Age-Related Changes in the Bone Marrow

**DOI:** 10.5812/iranjradiol.4494

**Published:** 2011-12-25

**Authors:** Ekrem Olcu, Mubeccel Arslan, Vedat Sabanciogullari, Ismail Salk

**Affiliations:** 1 Department of Radiology, Afsin State Hospital, Kahramanmaras, Turkey; 2 Department of Radiology, Cumhuriyet University, School of Medicine, Sivas, Turkey; 3 Department of Anatomy, Cumhuriyet University, School of Medicine, Sivas, Turkey

**Keywords:** Cranial Fossa, Posterior, Magnetic Resonance Imaging, Bone Marrow

## Abstract

**Background:**

The clivus is a bone region between dorsum cella and foramen magnum. It can be evaluated very clearly in routine brain magnetic resonance imaging (MRI) dueto its central location.

**Objectives:**

Quantitative and qualitative evaluation of the clivus and its changes according to age in a group of healthy people.

**Patients and Methods:**

The transition of clival bone marrow to fatty marrow by the increasein age is examined by MRI in 105 men and 105 women who had no clival and bone marrow pathology on MRI. The clivus/pons, clivus/CSF intensity values and clival bone marrow imaging patterns according to age groups were prospectively evaluated using a 1.5 Tesla MR device.

**Results:**

When age groups were individually compared, there were meaningful statistical differences both in men and women in terms of clivus/CSF and clivus/pons intensity ratios (both *Ps* < 0.05). Clivus/pons and clivus/CSF intensity ratios were found to be increased with age in all cases. The distribution of age groups according to stages in all individuals was statistically meaningful (P < 0.05). When the appearance patterns of both genders in every ten-fold age were examined, stage III bone marrow was observed more in elder ages.

**Conclusions:**

As a result, besides the fact that standard ranges determined for clivus/CSF, clivus/pons intensity ratios according to age may be used in the assessment of potential pathological cases involving bone marrow; they can also be leading in the diagnosis of bone marrow diseases when taken into consideration together with clinical and laboratory data.

## 1. Background

Clivus is the name given to the bony area located between foramen magnum and dorsum sella on the skull base. Thanks to its central location, it may be clearly evaluated in routine brain magnetic resonance imaging (MRI); since it is quite important for the diagnosis of primary neoplasms, metastatic tumors and hematopoietic diseases related to the clivus [1][2]. As with the other bones in the body, the active bone marrow functioning in blood cell production named as the red bone marrow is increasingly more exposed to the infiltration of fat cells with age and turns into inactive yellow bone marrow [3]. In this regard, the essential matter in the observation of bone marrow through MRI is the ratio between fat and hematopoietic tissue [4].

During visual evaluation, three separate bone marrow patterns have been observed in the study of clivus through MRI. As the bone marrow is rich in hematopoietic tissue in childhood, clivus is observed as homogeneously hypointense and this image is named as stage I. Due to aging, the infiltration of fat cells occurs in the bone marrow and an intermediary stage is seen. In this stage, a heterogeneous intensity in which there are partial red bone marrow and partial yellow bone marrow both in high and low intensity is seen. This stage is named as stage II. The image named as stage III is the homogeneous hyperintense image of the hypoactive bone marrow [2][3]. So far, studies have generally been conducted on the vertebra, femur and pelvis bones to evaluate the healthy and pathological qualities of bone marrow in MRI. Despite the fact that the skull is examined in most MR imaging units, there are only a few studies to evaluate the signal intensity in cranial bone marrow [4][5].

The clivus was preferred in our study due to the fact that it can easily be evaluated in routine brain MRI on account of its central location and exposure to the latest age-related changes [1][6].

## 2. Objectives

In brain MRI, by proportioning intensity values (signal intensity ratios of clivus/pons and clivus/CSF) measured from equal size areas at T1-weighted sequence at midsagittal sections, normal ranges have been determined for clivus intensity values according to age groups and gender.

## 3. Patients and Methods

### 3.1. Study Group

This study was conducted from May, 2005 to January, 2006 on 105 men and 105 women who applied to the MRI Unit of the Radiology Department of Cumhuriyet University of Medicine for a brain MRI examination, who had never had intracranial surgery before, who had never taken radiotherapy and chemotherapy, who had no known diseases of the bone marrow and whose MRI findings were evaluated as within normal ranges. The selected individuals were examined within seven age groups, in each of which there were 15 men and 15 women, making a total of 30 persons. The study has been approved by the Cumhuriyet university ethics committee.

### 3.2. Imaging Technique

MRI examination was conducted in a system with 1.5 Tesla machine (Excelart, Toshiba, Japan) using a standard head coil. Of the Spin Echo (SE) T1 weighted images taken on a transverse plane [repetition time (TR): 550 ms, echo time (TE): 15 ms, flip angle (FA): 70/180, section thickness: 5 mm, matrix: 160 × 256], the section passing from the midst of the cranium was chosen for examination. In this section, mid-structures such as the clivus, the pons and the fourth ventricle are observed on the same plane. Digital measurements of signal intensities were performed from the central places of the clivus, pons and the cerebrospinal fluid within the fourth ventricle and the air neighboring cranium (it represents background noise) which is at the same vertical plane as the clivus.

The shape and size of the limited area whose intensity measurement was done were determined according to software programs that allow the MRI system to carry out some actions on the image. Preferably the circular region of interest (ROI) was used and we tried to standardize the size of the area to be measured as 0.10 cm2. By choosing a reasonable size value, difficulty of evaluation in the pediatric age group due to smallness of the clivus area and the possibility of the existence of cortical bone in ROI in the adult age group were minimized.

### 3.3. Evaluation

3.3.1. Visual Evaluation

**Figure 1:**
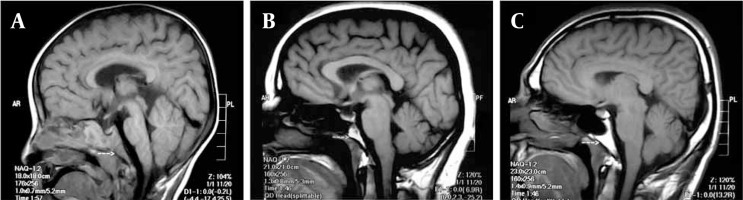
Qualitative evaluation of clivus bone marrow. A, Stage I image pattern of clivus bone marrow according to the qualitative evaluation in a 2-year-old boy; B, Stage II image pattern of clivus bone marrow according to the qualitative evaluation in a 42-year-old female individual; C, Stage III image pattern of clivus bone marrow according to the qualitative evaluation in a 34-year-old male individual. The arrows point to the clivus.

Signal intensity of the clivus was evaluated visually in T1 weighted MRI. In cases in which the clivus was observed to be homogeneously hypointense, it was accepted as stage I clivus bone marrow, in cases in which clivus was observed to have a heterogeneous intensity pattern in which there were both hypointense and hyperintense areas, it was accepted as stage II bone marrow and in cases in which clivus bone marrow was homogeneously hyperintense, it was accepted as stage III bone marrow (Figures 1 A-C).

3.3.2. Digital Evaluation

**Figure 2:**
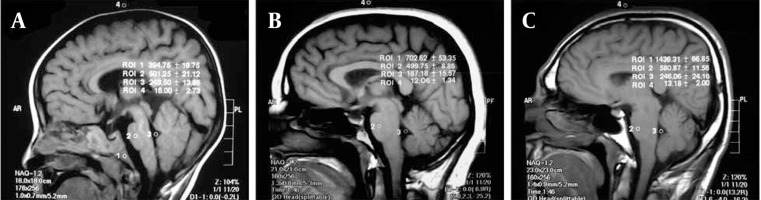
Quantitative assessment of clivus bone marrow signal intensity based measurements on MRI. A, In a 2-year-old boy; B, In a 42-year-old woman; C, In a 34-year-old man (1. Clivus, 2. Pons, 3. CSF, 4. Air)

The signal intensity measurement value found by using air in the vertical neighborhood of the cranium (which represents background noise) was subtracted from the signal intensity values measured from the clivus, pons and cerebrospinal fluid. Using these values, the ratios of clivus/pons and clivus/CSF were calculated. Signal intensity of the pons and cerebrospinal fluid were measured in order to ensure standardization (Figure 2 A-C).

### 3.4. Statistical Analysis 

For statistical analysis, Student’s t-test was used to compare the intensity ratios between genders; ANOVA and post-hoc Tukey tests were used to compare these ratios among the three qualitative grades. Age distribution between males and females was compared using the Fisher’s exact test. P < 0.05 was considered as statistically significant.

## 4. Results

Two-hundred ten individuals (mean age, 34.50 ± 20.42 years; range, 0-77 years) were enrolled in the study. One-hundred five of the individuals were men (mean age, 34.63 ± 20.46 years) and 105 were women (mean age, 34.37 ± 20.48 years) (P > 0.05).

Clivus/CSF and clivus/pons intensity ratios were compared in all age groups, no significant statistical difference was found between men and women (P > 0.05). However, when age groups were individually compared, there was significant statistical difference both in men and women in terms of clivus/CSF and clivus/pons intensity ratios (P = 0.001). It was seen that clivus/CSF and clivus/pons intensity ratios increase with age (Table 1).

**Table 1:** Descriptive Values of Clivus/CSF and Clivus/Pons Intensity Ratios Comparatively for Each Age Group in Men and Women

**Age, y**	**Clivus/CSF a, Mean ± SD**		**Clivus/Pons b, Mean ± SD**	
	**Female, (n = 105)**	**Male, (n = 105)**	**Female, (n = 105)**	**Male, (n = 105)**
0-9	2.18 ± 0.47	2.31 ± 0.71	0.91 ± 0.19	1.06 ± 0.22
10-19	2.97 ± 0.47	2.62 ± 0.39	1.24 ± 0.15	1.19 ± 0.14
20-29	3.04 ± 0.50	3.41 ± 0.66	1.30 ± 0.18	1.42 ± 0.19
30-39	3.37 ± 0.77	3.69 ± 0.69	1.42 ± 0.17	1.57 ± 0.23
40-49	3.56 ± 0.78	3.72 ± 0.51	1.54 ± 0.17	1.59 ± 0.20
50-59	3.96 ± 1.01	4.41 ± 1.00	1.72 ± 0.28	1.85 ± 0.30
≥ 60	4.63 ± 1.04	4.75 ± 1.17	1.74 ± 0.26	1.85 ± 0.37

^a^ F = 19.12; P = 0.001

^b^ F = 22.31; P = 0.001

The distribution of age groups according to stages in all individuals was statistically significant (P = 0.001). When the appearance patterns of both genders and all individuals in every ten-fold age were examined, stage III bone marrow was observed more in elder ages (Table 2).

**Table 2:** Distribution of Age Groups According to Stages in All Individuals a

**Age, y**	**Stage I, No. (%)**	**Stage II, No. (%)**	**Stage III, No. (%)**
0-9	22 (73.3)	8 (26.7)	0
10-19	8 (26.7)	16 (53.3)	6 (20)
20-29	0	22 (73.3)	8 (26.7)
30-39	1 (3.3)	16 (53.3)	13 (43.3)
40-49	1 (3.3)	11 (36.7)	18 (60)
50-59	0	9 (30)	21 (70)
≥ 60	0	6 (20)	24 (80)

^a^ P = 0.001

The association between the radiographic stage and clivus/CSF and also clivus/pons intensity ratios were significant (both Ps = 0.001). It was seen that clivus/CSF and clivus/pons intensity ratios increase with stage (Table 3).

**Table 3:** Descriptive Values of Clivus/CSF and Clivus/Pons Intensity Ratios in All Individuals According to Stages

	**Stage I, Mean ± SD, (n = 32)**	**Stage II, Mean ± SD, (n = 88)**	**Stage III, Mean ± SD, (n = 90)**	***P* value**
Clivus/CSF	2.34 ± 0.70	3.30 ± 0.78	4.01 ± 1.03	0.001
Clivus/Pons	1.00 ± 0.23	1.40 ± 0.25	1.68 ± 0.31	0.001

## 5. Discussion

The signal intensity of the bone marrow depends upon the amount of fat, water and cells. As the fat content with its different intensity value may clearly be noticed in T1 weighted images, MR imaging is a suitable method in bone marrow studies. Change in the normal signal intensity may be the sole sign of a systemic disease in the early stages or even earlier. During birth, red bone marrow is dominant both in the appendicular and axial skeleton [4].

Transformation from active bone marrow named as red bone marrow into yellow bone marrow starts primarily from the appendicular skeleton and goes on through the axial skeleton; as for the long bones, the process starts at the diaphysis and goes on through the metaphysis [4][7][8]. This transformation may clearly be seen on T1 weighted images. While low signal intensity is taken from the red bone marrow rich in active hematopoietic tissue, high signal intensity is taken from the yellow bone marrow whose fat content has increased [1][9][10].

In the subjective evaluation of MR images, the hypointense red bone marrow image of childhood is named as stage I; the image involving both hypointense and hyperintense areas due to increase in age is named as stage II; the image of hyperintense bone marrow observed during adulthood is named as stage III [2][3].

Dawson et al. [11] evaluated the pelvis bone marrow distribution according to age on individuals aged 0-24 using MRI and found that signal intensity increased in all areas proportionally to age except for the acetabulum and there was heterogeneous bone marrow pattern in the acetabulum in all ages. They verified the results of this study by means of comparing them to the preparations made out of the microscopic findings of bone marrow biopsy from cadavers.

Moore and Dawson [8] determined the change of red and yellow bone marrow regionally at the femur according to age by means of MRI. They established the importance of knowing the bone marrow pattern that may be accepted as normal within the same group by examining six groups consisting of 0-1, 1-5, 6-10, 11-15, 16-20 and 21-24 years age groups. Furthermore, similar to our study, they maintained that there was no meaningful statistical difference between men and women in terms of the change in bone marrow due to age.

Zawin and Jaramillo [7] showed that bone marrow intensity increased with age on MRI in the humerus, sternum and clavicle by classifying individuals as under 1 year old, 1-5, 6-10, 11-15 and over 15 years old and compared their findings with previously conducted studies. According to these, while conversion to fatty bone marrow at the humerus diaphysis is completed by the age of 6 years, conversion has been said to be completed by the ages of 12-14 years in anatomical studies. Besides, while anatomical studies have established that conversion at the distal metaphysis starts by the age of 16-18 years, Zawin and Jaramillo stated that they observed conversion at the distal metaphysis in children under age 1 and this conversion is completed by the age of 11-15 years.

Hajek et al. [12] showed focal fatty infiltration areas with a diameter of 0.5-1.5 cm mostly on the areas close to vertebral plates and posterior elements on the vertebral column on MRI at T1 and T2 weighted sequences and they maintained that this increases with age. They supported their study by comparing histopathological findings at the focal fatty infiltration areas in anatomic cadaver samples taken from three fresh cadavers, to MRI findings. Determining focal fatty infiltration areas due to age, they also stated that this finding should not be confused with other pathologies such as spondilitis, spondylosis, kyphoscoliosis and intraosseous lipoma.

Simonson and Kao [6] examined the normal signal pattern of cranial bone marrow on the sagittal T1 weighted MRI of 324 subjects aged 0-18 in their retrospective studies including the last seven years. They evaluated the conversion from red bone marrow into fatty bone marrow in four stages by making a visual comparison with fat and muscle tissue. Performing this evaluation on the different parts of the cranium, they found different results according to age on areas they examined. They stated that conversion takes place on face bones earlier than calvarial ones and at the same time, bone marrow in pneumatized bones should be isointense with fat before pneumatization.

Simonson and Kao [6] found that the basio-occipital part of the clivus is the place which is exposed to the latest conversion. They said that they observed all kinds of signal intensities in this basio-occipital part in individuals aged 2-13 years and that homogeneous fatty marrow intensity increases with age between ages 2 and 18 years. Moreover, they expressed that bone marrow in the basio-occipital becomes isointense with fat approximately during ages 11.9 ± 0.24 years. However, in our study stage III bone marrow having an isointense appearance with fat in the 0-9 years age group was not observed and it was only observed at a rate of 53.3% in the 10-19 years age group (Table 2).

Simonson and Kao [6] stated that there is no difference between genders in terms of bone marrow conversion and bone marrow is exposed to conversion with age.

Okada et al. [5] evaluated the normal conversion of cranial bone marrow retrospectively in 246 individuals under 25 years of age through MRI. They alleged that stage I bone marrow pattern was on no account observed after age 6 in the clivus area. Furthermore, they reported that stage II bone marrow pattern was seen in ages 3-4 with a ratio of 68-70% and the earliest stage that stage III bone marrow pattern emerges is the age of 2 and more than 90% of the individuals had stage III bone marrow pattern at the age of 15. Based on this data, they maintained that if stage I bone marrow pattern was observed on the MRIs of individuals aged 3-6, this should be evaluated on behalf of abnormal cell proliferation; and if stage I or stage II bone marrow pattern was observed in individuals over 15, searching for a pathology beneath this would be right.

In our study, stage I bone marrow pattern in individuals in the 10-19 age group has been found as 26.7%. Stage III bone marrow pattern has not been observed in individuals at the age group 0-9 years and it could only be seen at a rate of 80% in individuals over 60 (Table 2). These findings do not comply with Okada et al.’s findings. The similarity of our study to this one is that it has been clearly established that cranial bone marrow indicates a change from low intensity to high intensity according to age. In our study, both intensity ratios of clivus bone marrow and the number of individuals in the advanced stage increased with age (P = 0.001, Table 1, 2 & 3).

In their retrospective study, Kimura et al. [2] evaluated clivus bone marrow intensities of 330 individuals aged 20-88 years on their T1 weighted midsagittal images in three stages by comparing them visually to pons and subcutaneous fat intensities. It was alleged in the study that stages I, II, and III bone marrow pattern were found to be at a ratio of 1/3 between ages 20-29 years and stage II bone marrow pattern made a rapid increase in the 40 to 49 years age group.

In our study, stage I marrow pattern has not been come across between ages 20 and 29, stage II marrow pattern has been found as 73.3% and stage III marrow pattern has been found as 26.7% (Table 2). When these values are compared to Kimura et al.’s study [2], besides the fact that stage I marrow pattern is observed between the 20 and 29 years ages, a striking increase draws attention in stage II marrow pattern. In addition, in our study as being different from Kimura’s findings in the 20-29 and 30-39 age groups, a rapid increase is noticed in stage II marrow pattern. As being different from the form of increasing in stage III bone marrow pattern in age group 20-29 and following ages in Kimura et al.’s study [2], in our study a rapid increase in stage III bone marrow pattern in the 40 to 49 age group and the continuation of this increase in the following ages is noticeable (Table 2).

Besides Kimura et al. [2], Oyar et al. [3] too evaluated the normal bone marrow pattern of clivus on T1 weighted midsagittal images in three stages in 260 individuals aged 0-79 years. While in Oyar et al.’s study [3] stage I bone marrow pattern was found to be 96% in individuals aged 0-9 and stage II bone marrow pattern was found to be 4% and stage III bone marrow pattern was not observed in this age group, in our study, stage III bone marrow pattern was not observed in this age group either; stage I bone marrow pattern was found to be 73.3% and stage II bone marrow pattern was found to be 26.7% (Table 2).

Oyar et al. [3] alleged that stage III bone marrow pattern beginning with the ages 10-19 and the ratio increased in every decade. They also alleged that stage I bone marrow pattern indicated a rapid decrease in the second decade and it was on no account observed at the age of 50 and after. These findings by Oyar et al. [3] comply with the findings of our study. As well as evaluating clivus in terms of the appearance pattern similarly to Oyar et al. study’s [3], our study also carries the purpose of determining bone marrow intensity according to age group in digital ranges (Table 1). Bayramoglu et al. [13] applied qualitative and quantitative examination together by means of proportioning digital intensity values to visual assessment. In the prospective study on 201 individuals whose brain MRIs had no pathology, they determined bone marrow intensity ratios according to age groups. They found stage I bone marrow ratio as 91.7% in age group 0-9 and stage II bone marrow ratio as 46.2% in the 20 to 29 years age group. However, in our study stage I bone marrow ratio was found to be 73.3% in the 0-9 years age group and stage II bone marrow ratio was found to be 73.3% in the 20-29 years age group. As for other age groups, results close to Bayramoglu et al.’s [13] were obtained (Table 2).

Bayramoglu et al. [13] established that clivus bone marrow intensity ratios were statistically higher at a meaningful degree in men than women and they also stated that this may be due to the fact that the mineral content of bone marrow is different in women due to sex hormones. Despite this, in our study no meaningful difference was found between men and women in terms of the averages of clivus/CSF and clivus/pons intensity ratios and even when the age groups were individually compared (Table 1).

As a result, it has been found in the comparison of stages with intensity ratios that the averages of intensity ratios in each of the three stages are different (Table 3). However, even if their averages are different from one another, it has been seen that individuals having similar intensity ratios may be evaluated as both stage II and stage III. Therefore, besides the fact that standard ranges determined for clivus/CSF and clivus/pons intensity ratios according to age can be used in the assessment of potential pathological cases involving bone marrow; they may also be leading in the diagnosis of bone marrow diseases when taken into consideration together with clinical and laboratory data.
